# Genetic variability and spatial distribution in small geographic scale of *Aedes aegypti* (Diptera: Culicidae) under different climatic conditions in Northeastern Brazil

**DOI:** 10.1186/s13071-016-1814-9

**Published:** 2016-10-04

**Authors:** Lizandra Makowski Steffler, Silvio Santana Dolabella, Paulo Eduardo Martins Ribolla, Carine Spenassatto Dreyer, Edilson Divino Araújo, Rosane Gomes Oliveira, Walter Fabrício Silva Martins, Roseli La Corte

**Affiliations:** 1 Programa de Pós-Graduação em Biologia Parasitária, Universidade Federal de Sergipe, Av. Mal Rondon s/n, CEP 49.100-000 São Cristóvão, Sergipe Brazil; 2 Departamento de Morfologia, Universidade Federal de Sergipe, Av. Mal Rondon s/n, CEP 49.100-000 São Cristóvão, Sergipe Brazil; 3 Departamento de Parasitologia, Universidade Estadual Júlio de Mesquita Filho, Rubião Junior, CP 510, CEP 18618-900 Botucatu, São Paulo Brazil; 4 Departamento de Biologia, Universidade Federal de Sergipe, Av. Mal Rondon s/n, CEP 49.100-000 São Cristóvão, Sergipe Brazil; 5 Programa de Pós-Graduação em Biotecnologia, Rede Nordeste de Biotecnologia, Universidade Federal de Sergipe, Av. Mal Rondon s/n, CEP 49.100-000 São Cristóvão, Sergipe Brazil; 6 Departamento de Biologia/CCBS, Universidade Estadual da Paraíba, R. Baraúnas 351, Campina Grande, CEP 58.429-500 Paraíba Brasil

**Keywords:** ISSR-PCR, Single nucleotide polymorphism, Entomological surveillance, Vector control, Zika, Dengue, Chikungunya, Arboviruses

## Abstract

**Background:**

The study of the genetic structure of *Aedes aegypti* is essential to understanding their population dynamics as well as for the analysis of factors responsible for their resistance and ecological adaptation. The use of molecular markers in identifying differences amongst populations of *Ae. aegypti* in different geographical areas as well as the temporal variation of the vector populations has contributed to the improvement of vector control strategies. The present study aims to determine the genetic variability of *Ae. aegypti* populations in a small geographical area (state of Sergipe, Northeastern Brazil) by means of inter-simple sequence repeat (ISSR) and single nucleotide polymorphism (SNP) molecular markers.

**Results:**

ISSR markers revealed a more heterogeneous pattern of genetic diversity among the populations with an expected heterozygosity (*H*_E_) ranging from 0.261 ± 0.03 to 0.120 ± 0.032, while a similar trend was detected through SNPs across populations with an *H*_E_ between 0.375 ± 0.054 and 0.269 ± 0.042. The population’s genetic differentiation assessed with ISSR and SNP markers indicated a very low structuring among the populations with the highest diversity observed within the populations 72 % (ISSR) and 92 % (SNP). Clustering analysis also suggested little variation among populations: the seven populations were grouped into only three ISSR clusters and a single panmictic group based on SNP markers. The present study identified a close relationship between the populations, which probably results mainly from passive gene flow between mosquitoes from distinct geographic regions, influenced by humans commuting along roads.

**Conclusions:**

There was an intense migration of mosquitos across municipalities, leading to a potential increase in risk of arbovirus and insecticide resistance associated-alleles spreading between mosquito populations.

**Electronic supplementary material:**

The online version of this article (doi:10.1186/s13071-016-1814-9) contains supplementary material, which is available to authorized users.

## Background

*Aedes aegypti* is a major vector of several human arboviruses including Yellow fever, Dengue fever, Chikungunya and Zika virus in several tropical and sub-tropical countries. In Brazil, the mosquito has been reported in all states and the number of cities where it is found has increased with time [1]. The prevalence of this vector is alarming as it is the main factor related to Dengue fever, Zika and Chikungunya epidemics. It also reveals the failure of the current vector control strategy held by the National Dengue Control Program, based on breeding site elimination, which has prompted the search for new control strategies [2–4].

The discovery of genetic markers has facilitated the following-up of groups of genes or of genome segments related to a phenotype of interest. Studies based on biological markers such as isoenzymes, random amplified polymorphic DNA (RAPD), restriction fragment length polymorphism (RFLP), single-strand conformation polymorphism (SSCP), single nucleotide polymorphism (SNPs) and microsatellites have resulted in the construction of genetic maps for *Ae. aegypti* [5]. In spite of this, a limited number of new microsatellites have been identified in *Ae. aegypti* [6–9]. Studies on new microsatellite loci in *Anopheles gambiae*, *Culex quinquefasciatus* and *Ae. aegypti* have shown they are more abundant (with regards to the genome frequency percentage) in *An. gambiae* (0.75 %) and less abundant in *Ae. aegypti* (0.109 %) [10].

The inter-simple sequence repeat-polymerase chain reaction (ISSR-PCR) technique amplifies regions between microsatellites, using core microsatellite sequences as primers. Two important characteristics are observed when using ISSR-PCR: (i) high levels of polymorphism, which is particularly important for geographically small-scale intraspecific studies that aim to genetically identify different populations of a species; and (ii) the increased annealing temperature of the primers, which leads to high reproducibility of the DNA band pattern [11].

Understanding the genetics of *Ae. aegypti* is essential in order to gain an in-depth understanding of population dynamics, factors responsible for resistance to insecticides and ecological adaptation [12]. Molecular markers can aid the identification of geographical and temporal differences amongst *Ae. aegypti* populations, contributing to the improvement of mosquito vector control strategies [13–15]. Investigation of the *Ae. aegyti* genome indicates that SNP frequency is often dependent on gene function [16]. The partial sequencing of seven *Ae. aegypti* genes revealed the existence of 53 polymorphic SNPs, eight of these were used to outline the population structure of three *Ae. aegypti* populations, proving to be highly polymorphic markers that are useful for population studies [17].

The aim of the present study was to determine the genetic variability of *Ae. aegypti* populations on a small geographical scale with high spatial heterogeneity by means of SNP and ISSR molecular markers. The study included cities located in humid tropical, agreste and semiarid areas in the state of Sergipe, northeastern Brazil. The results of this study will not only contribute to the vector biology knowledge base, but may even be suitable for supporting new approaches of mosquito control, and furthering studies of the main factors related to the genetic differences observed.

## Methods

### Mosquito samples and DNA extraction

The study was carried out in the state of Sergipe, northeastern Brazil (9°31′S to 11°33′S and 36°25′W to 38°14′W), covering an area of 21,918 km^2^ and including 2,068,017 inhabitants [18]. The distribution of annual precipitation in this state is highly heterogeneous, with a decreasing gradient from the shoreline’s humid tropical climate and annual precipitation of over 1600 mm, stretching through to the agreste (transition zone) towards the sertão (semiarid climate), where annual precipitation below 500 mm has been recorded. The rainy period in this state is concentrated between April and August, with peaks in May, June and July [19].

**Fig. 1:**
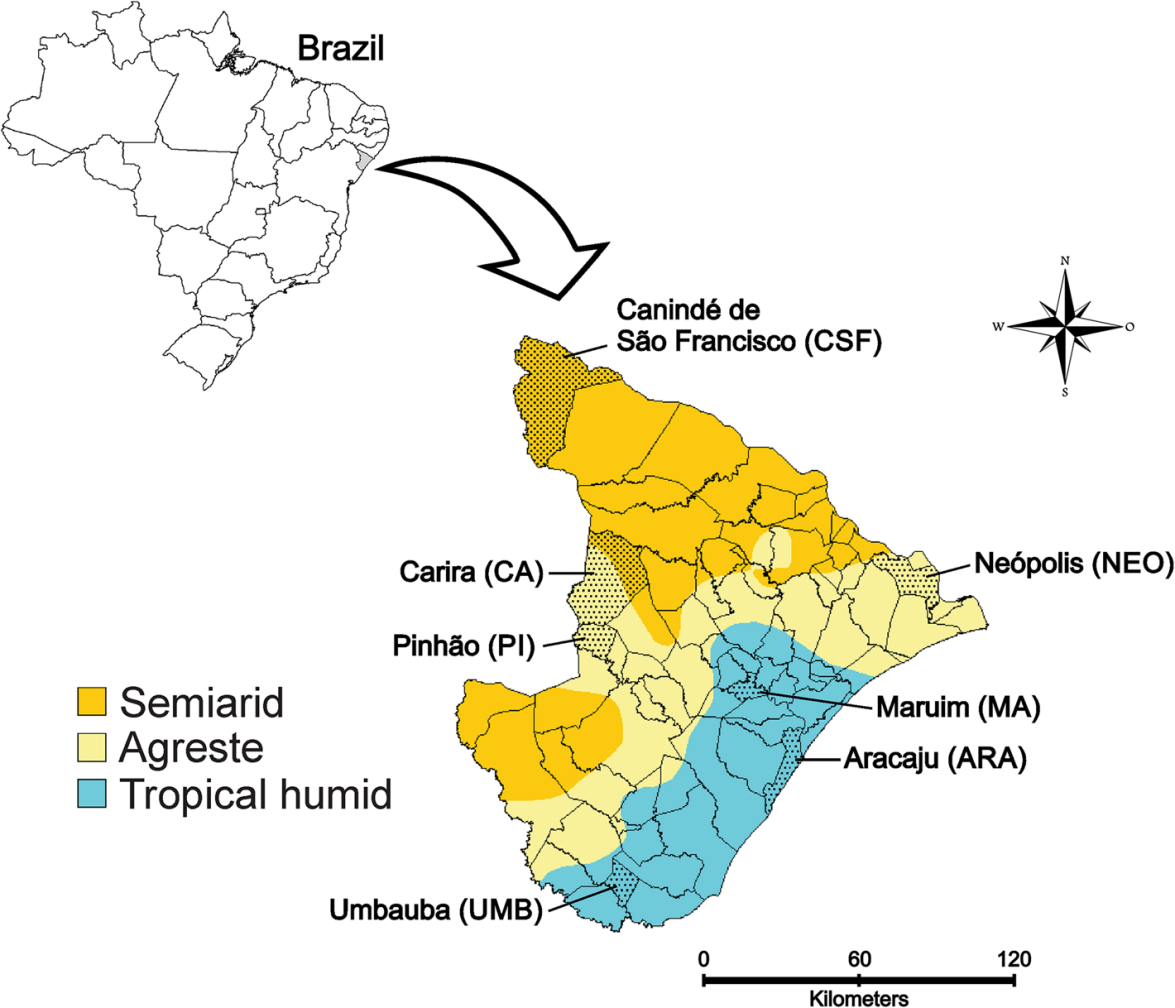
Map of the state of Sergipe (Brazil) showing the locations of the cities selected for the study

*Aedes aegypti* was sampled from 7 locations in Sergipe, including Carira (CA), Pinhão (PI) and Neópolis (NEO) (Agreste), Canindé de São Francisco (CSF) (semiarid), Maruim (MA), Aracaju (ARA) and Umbaúba (UMB) (humid tropical) (Fig. 1). A hundred ovitraps were installed in each city, except the state capital Aracaju, where only one neighborhood was sampled with 30 ovitraps. The selection of the cities aimed to include most of the state, taking into consideration the different climatic types (humid tropical, agreste and semiarid). The longest distance registered was between the cities of Canindé de São Francisco and Umbaúba (road = 243 km/linear = 193 km) and the shortest one was between Carira and Pinhão (road = 30 km/linear = 20 km). Although Carira and Pinhão are geographically very close, they were treated as distinct populations since they showed different levels of resistance to temephos (data not shown) in the susceptibility test performed for these populations in our laboratory.

Rearing of the *Ae. aegypti* populations was held in an acclimated area with monitored temperature and humidity (temperature at 26 °C ± 2 °C, air relative humidity at 70 % ± 20 °C and 12-h photoperiod). For each city, 20 three-day-old *Ae. aegypti* females from the wild population were analyzed, adding up to a total of 140 individuals. The genomic DNA was extracted from the whole body according to a published method [20], and stored at -20 °C.

### Genetic analysis

#### ISSR genotyping

Two primers designated (CA)_8_RY and (GA)_8_RY were used. The primers possess one random purine (R) and pyrimidine (Y) anchored in the 3′ portion. The premix for PCR consisted of a reaction of 15 μl, containing 50 ng genomic DNA, RB 10× buffer (10 mM Tris-HCl pH 8.5, 50 mM KCl, 1.5 mM MgCl_2_), 1.5 U of Taq polymerase (5U/μl) (Invitrogen, Carlsbad, CA, USA), 0.2 mM of dNTPs and 1 μM of the primers (CA)_8_RY or (GA)_8_RY. Samples were processed in duplicate as follows: denaturation at 95 °C for 5 min, followed by 39 cycles of denaturation at 95 °C for 30 s, annealing at 47.5 °C and 45.2 °C for 30 s for the primer (CA)_8_RY and (GA)_8_RY, respectively, and extension at 72 °C for 2 min. A final extension step at 72 °C for 5 min was also included. The amplification product underwent 1 % agarose gel electrophoresis using ethidium bromide as colorant. The selection of the bands was carried out with the aid of the GelAnalyser v. 2010a software (http://www.gelanalyzer.com) and reproducibility of the gel comparison results obtained from the amplification in duplicate, with each band considered as a locus.

#### SNPs genotyping

For the characterization of populations, we used 9 SNP markers related to the genes *ef2* (elongation factor), *aeimuc1* (mucin-like protein), *nak* (sodium/potassium channel), *pgk* (phosphoglycerate kinase), *apolp-2* (apolipophorin II), *ferh* (ferritin heavy chain), *cyp9j2* (cytocrome P450), *tsf* (transferrin) and *chym* (chymotrypsin), which have previously been selected for the population study of *Ae. aegypti* in Brazil [17]. These genes were chosen because they are randomly distributed in the *Ae. aegypti* genome.

The DNA of each mosquito underwent amplification by quantitative real-time PCR using the StepOnePlus™ v. 2.1 platform (Thermo Fisher Scientific, Waltham, MA, USA) using 96-well optical plate and TaqMan® system (Thermo Fisher Scientific, Waltham, MA, USA) for the allelic discrimination. The initial amplification conditions were 25 °C for 30 s (equipment pre-reading); denaturation at 95 °C for 3 min, followed by 40 cycles of denaturation at 95 °C for 3 s, annealing/extension at 60 °C for 30 s and a final step at 25 °C for 30 s. SNPs were automatically identified with the help of the StepOnePlus™ v. 2.1 platform software (Thermo Fisher Scientific, Waltham, MA, USA).

### Data analysis

The index of genetic diversity and the molecular variance (AMOVA) were carried out using *GenAlEx* [21]. Genetic differentiation among populations was estimated through a Bayesian analysis using STRUCTURE 2.3 [22]. In this analysis, all 140 individuals were probabilistically assigned to a single cluster without using the known geographic sample collection location. To identify the optimal number of clusters (*K*), with the assumption that the sampled belong to an unknown number of *K* genetically distinct clusters, twenty independent runs were conducted for each *K* value (ranging from *K* = 1 to *K* = 10), with 5000 interactions and 50,000 replications. The most likely *K* value was calculated for each run with the log likelihood [LnP(D)] method and results compiled using CLUMPP [23]. Population structure was also evaluated with discriminant analysis of principal components (DAPC) using the adegenet R package [24]. To identify an optimal number of clusters for the DAPC clustering, K-means values were sequentially tested and then compared using Bayesian information criterion (BIC), with the lowest value of BIC used as the likely number of population clusters.

## Results

### Genetic diversity

#### ISSR markers

**Fig. 2:**
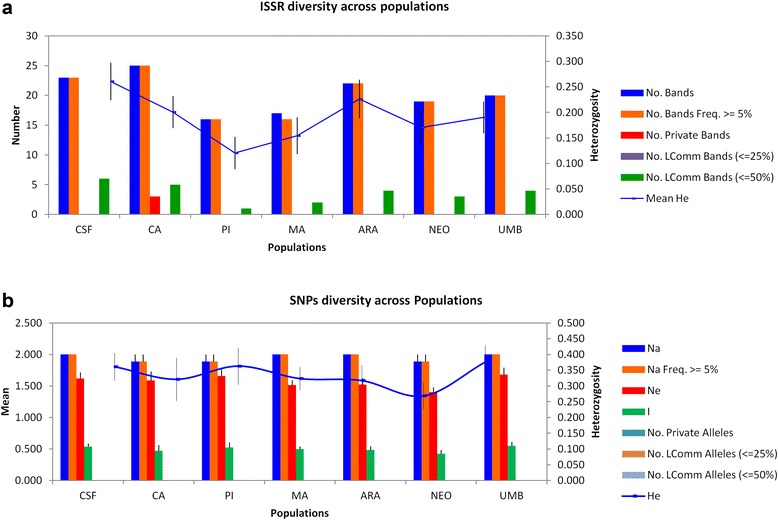
Characterization of genetic diversity in *Aedes aegypti* mosquitoes from Sergipe, Brazil. **a** and **b** correspond to genetic diversity index based on ISSR and SNPs, respectively. CSF: Canindé de São Francisco; CA: Carira; PI: Pinhão; MA: Maruim; ARA: Aracaju; NEO: Neópolis; UMB: Umbaúba; No. Bands: No. of different b ands; No. private bands: No. of bands unique to a single population; No. LComm Bands: No. of locally common bands; Na: No. of different alleles; Ne: No. of effective alleles; I: Shannon’s information index; He: expected heterozygosity

For this analysis, 17 bands were selected for the primer (CA)_8_RY, which varied between 450 and 2230 base pairs (bp) and 13 bands for the primer (GA)_8_RY, which varied between 350 and 1700 bp, resulting in a total of 30 bands (Additional file 1: Table S1; Additional file 2: Figure S1), of which 29 were polymorphic. The highest number of bands was observed in the population CA with a total of 25 bands (with 3 private alleles), while the lowest number of bands was detected in NEO with only 19 markers (Fig. 2). Across populations, a significant difference was detected in the expected heterozygosity which ranged from *H*_E_ = 0.261 ± 0.03 to *H*_E_ = 0.120 ± 0.032 (Fig. 2 and Additional file 1: Table S1), with the percentage of polymorphic loci varying between 73.33 and 43.33 % in mosquitoes from CSF and PI, respectively.

### SNP markers

Across all populations, the 7 loci studied were polymorphic in 95.25 % of the locus/locations, with the exception of 3 loci; *pgk*, *nak* and *ferh* which were monomorphic in CA, PI and NEO, respectively (Additional file 3: Table S2; Additional file 4: Figure S2). Among the 7 loci, a higher genetic diversity was observed for *ferh* (*H*_*E*_ *=* 0.750) in PI where a distinct pattern of *H*_*E*_ was detected between populations (Additional file 3: Table S2, Additional file 5: Table S3). Amongst the populations, a similar index of genetic variability (Fig. 2) was observed, with the observed heterozygosis ranging from 0.375 ± 0.054 to 0.269 ± 0.042 in samples from PI and NEO, respectively.

In all 60 instances where polymorphic locus/populations were tested for locus Hardy-Weinberg (HW) equilibrium, significant deviation from HW equilibrium was not detected, with *P* > 0.05 (Additional file 5: Table S3). Nevertheless, for 6 loci, a significant departure from HW was observed, with *P* < 0.01; *apolp-2* (CA), *ferh* (PI), *nak* and *chym* (NEO) and *ef2* and *aelmuc1* (UMB). In all 6 loci, a deficit of heterozygosity was observed (Additional file 3: Table S2; Additional file 6: Tables S4 and S5).

### Genetic structure

The populations’ genetic differentiation was assessed using ISSR and SNP markers across all 7 populations. The genetic distances among populations ranged from 0.033 (NEO and MA) to 0.120 (ARA and UMB) for ISSR markers (Additional file 7: Figure S3), while for SNPs (Additional file 7: Figure S3), the highest genetic distance (0.145) was observed between PI and NEO and the lowest (0.008) between MA and ARA. Through AMOVA, the highest diversity within the population, 72 % (ISSR) and 92 % (SNP), was observed for both markers. In population and geographical clusters, a very low differentiation of 0 and 28 % for ISSR was detected, and 4 % in each category for SNP markers.

**Fig. 3:**
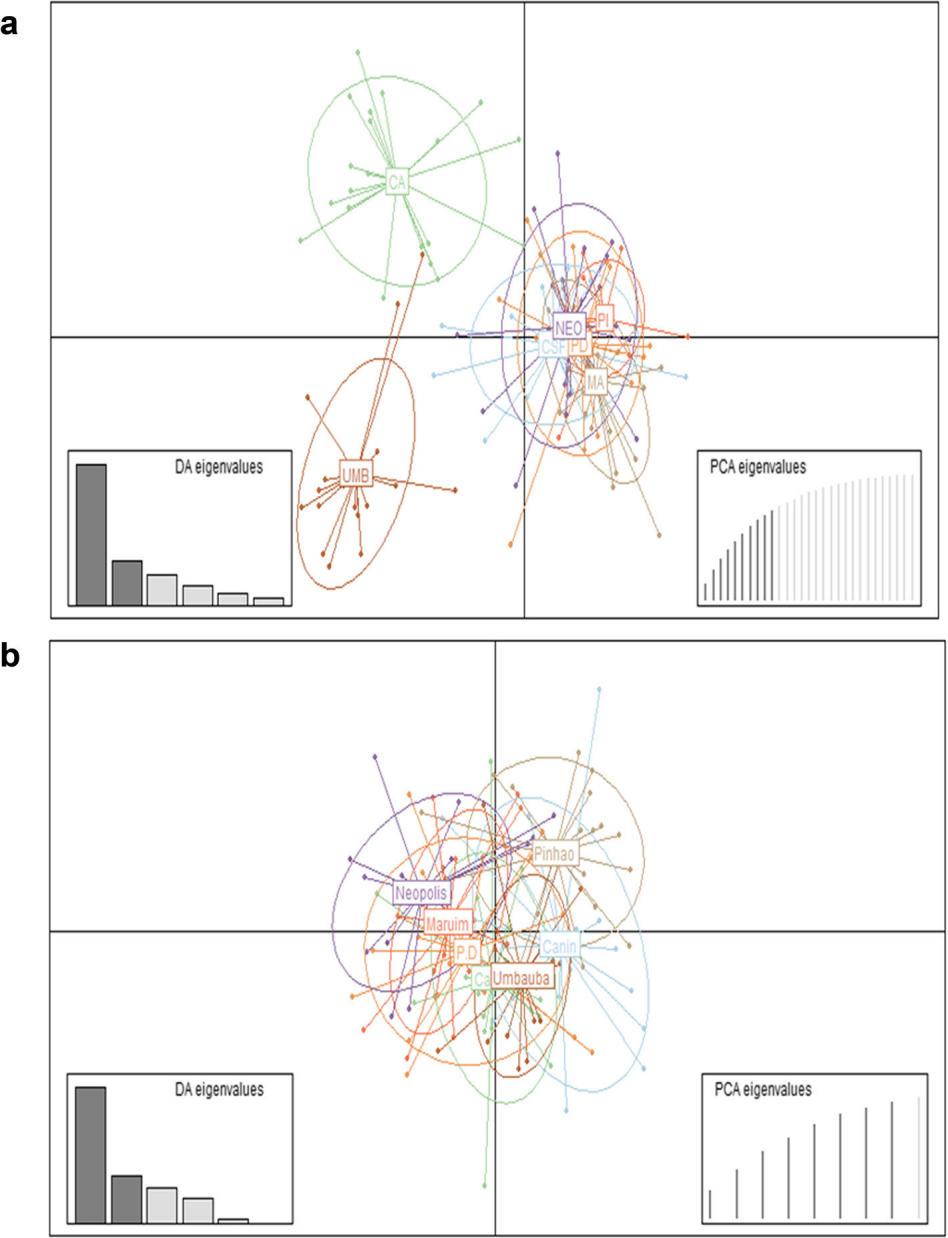
Genetic differentiation estimates among the seven *Ae. aegypti* populations from Sergipe, Brazil. **a**, **b** First and second Principal Components of the DAPC using ISSR and SNP markers, respectively. Inferred populations clusters are indicated by ellipses, which model 95 % of the corresponding variability. CSF: Canindé de São Francisco; CA: Carira; PI: Pinhão; MA: Maruim; ARA: Aracaju; NEO: Neópolis; UMB: Umbaúba

**Fig. 4:**
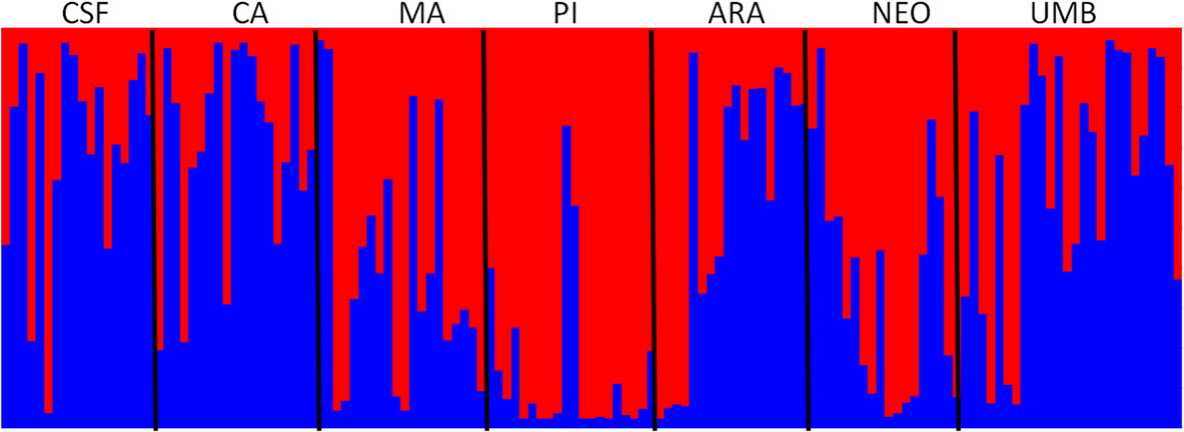
Bayesian cluster analysis based on ISSR markers. Diagrammatic representation of population clusters for the most likely *K* (*K* = 2), where each vertical bar represents an individual and each colour represents the probability of belonging to one of the two clusters from Bayesian STRUCTURE analyses. CSF: Canindé de São Francisco; CA: Carira, PI: Pinhão; MA: Maruim; ARA: Aracaju; NEO: Neópolis; UMB: Umbaúba

The small genetic differentiation suggested by AMOVA analysis was also suggested by DAPC and STRUCTURE analysis, indicating that the populations studied are very close related. For ISSR markers, DAPC and STRUCTURE analysis indicated the presence of only 2 and 3 clusters, respectively (Figs. 3a and 4). On the other hand, when using the SNP markers, both methods indicated an absence of differentiation among populations: all populations were grouped within a single cluster by DAPC (Fig. 3b) and had a very low and unstable delta *K*-value through the STRUCTURE analysis (Additional file 8: Figure S4). For both markers, no significant association between genetic and geographical distance was observed using the Mantel test (Additional file 9: Figure S5).

## Discussion

Based on both genetic markers, ISSR and SNPs a high level of genetic diversity was identified, with most of the markers in all seven populations analyzed being polymorphic. Nevertheless, our analysis indicated a different diversity trend across populations: with ISSR markers significant genetic variability was observed whereas with SNPs, no significant variation among the locations was observed. This distinct pattern of diversity was somewhat expected and could reflect their genetic properties, as ISSR is dominant and can amplify coding or neutral genomic regions while SNPs are located within specific expressed loci, which are more conserved and stable genomic regions. As such, the ISSR markers located in neutral genomic regions could be more diverse and reflect the populations’ recent dynamic events, such as variations in the effective population size, bottle necks and migration. Many studies have shown large populations experience more mutations than those with a small effective population size [25], while bottle necks (reductions in effective population size) reduce most-recent common ancestor (MRCA) alleles, which in turn reduces the average level of variability [26].

The genetic variation detected by ISSR markers might also be linked to the different climate characteristics that vary strongly within the small geographical area of Sergipe State. In this region, the climate is characterized tropical humid, agreste and semiarid. Rainfall and relative humidity across these regions ranges from 1355 mm to 700 mm and from 80 to 65 %, respectively [27]. Ecological studies of *Ae. aegytpi* populations from Paraíba State, Brazil, showed that, under experimental conditions with temperatures ranging from 25 to 32 °C, variation in climatic conditions was reflected in changes to mosquito larval development, longevity, fecundity, eggs quantity and body size [28, 29]. Consequently, the genetic variation observed among Sergipe’s populations could be linked to the ecological zone from where samples were collected.

It is also possible that the distinct genetic diversity between the populations might be associated with differences in predominance/availability of breeding sites in each city, i.e. predominance of laundry sinks in Aracaju [30] and water tanks (cisterns) in Pinhão. The increased availability of these breeding sites could result in distinct mosquito population densities and inbreeding, and then directly impact a population’s genetic diversity.

On the other hand, the similar genetic diversity observed with SNP markers across the populations/geographic groups might reflect the markers’ location in the coding regions of the genes analyzed, resulting in a lower mutation rate. It is also possible that these markers are behaving as neutral markers since they might not be linked with the possible selection pressure imposed by vector control interventions. If selection pressure was acting on one of these loci, a significant difference in allele frequency among populations would be expected due to, for example, a positive selection or selective sweep, which changes allele frequency by increasing the frequency of derived alleles and then increasing a population’s differentiation [31, 32]. For instance, the identification of selection footprints in insecticide resistance-associated alleles has been detected by identifying a reduction in genetic diversity [33, 34].

Even though most of the locus/population were under HW equilibrium, six markers (*apolp-2*, *ferh*, *chym*, *nak*, *ef2* and *aelmuc1*) were not in HW equilibrium in one population each. Since HW disequilibrium did not occur in all population/locus, the possibility of null alleles due to problems in the annealing sites of the initiators used was discarded [35]. Nonetheless, these deviations in HW’s equilibrium may be due to environmental differences among the populations’ geographic location. For instance, although the geographic dimension evaluated here is small, it presents the relevant climatic differences such as a long drought period and high temperatures, which are characteristic of the semiarid region. In particular, the deviation from HW for the *nak* loci in Neopolis and monomorphism in Pinhão is especially relevant as it is located in the Sodium channel gene, which is the target-site gene for pyrethroids and organochlorines insecticides [36]. The link between a possible selection sweep and this departure from HW equilibrium in these two populations has already been shown in other vector species such as *Anopheles* and *Culex* populations under insecticide selection pressure [37–39].

Both ISSR and SNP markers also indicated a high level of genetic similarity among populations, with the ISSR of seven populations grouped into three clusters whereas for SNPs, a single panmictic population was detected. Although both markers are in agreement with low differentiation among populations, the discordance between the number of clusters could result from inherent differences (dominant *vs* codominant) and level of polymorphism and mutation rates (i.e. most SNPs are biallelic in contrast to the multiallelic properties of ISSR due to slippage mutation processes) [40]. Thus, these features could result in distinct heterozygosities and allele frequencies, which may have implications in the pattern of population relatedness observed [41]. Secondly, the number of markers analyzed by ISSR was around four times higher than the number of SNPs investigated indicating that due to a close relationship between the populations, a larger number of SNP markers might be required to detect the populations’ differentiation using this marker.

The low differentiation in Sergipe’s cities observed by molecular variance analysis (AMOVA) for the ISSR and SNP markers, with most of the variation detected within the populations, was also reported in *Ae. aegypti* in northeastern Argentina and Uruguay. Around 89 % of the diversity observed within populations in those studies was based on the same markers applied here [11]. Furthermore, a similar pattern of differentiation as Sergipe’s population was also reported in mitochondrial DNA analysis which indicated the presence of only two haplotype groups of *Ae. aegypti* circulating in Brazil [42, 43]. Nevertheless, the subpopulations reported in this study for the ISSR marker with three population clusters might not represent the two previous clusters described for mitochondrial DNA, since it has been reported that populations can have the same mitochondrial haplotype but be distinct when analyzed with nuclear SNPs [17, 44].

In addition to the likely impact of the introduction of *Ae. aegypti* with little genetic variation in Brazil as suggested by mitochondrial markers [42], it is also important to consider that the differentiation among Sergipe’s population could reflect the intense gene flow mediated by the passive dispersion of mosquitoes, due to the intense traffic of vehicles between the cities. Our cluster analysis based on ISSR markers (Fig. 3a) also supports evidence of the possible impact of geographical distribution and route connection on a population’s differentiation as the two more distinct clusters: CA and UMB correspond exactly to the most distant mosquito collection points, separated by 243 km (Fig. 1).

Paduan et al. [44] showed that regions connected by trade routes tend to have a lower genetic differentiation among *Ae. aegypti* populations when compared to areas that are more isolated. Additionally, Damal et al. [45] suggest that limited urbanization does not represent a strong enough barrier to stop the genetic flow. This suggests that passive movement has great importance in the mosquito’s dispersion [46], as seen in our work, where road transportation may play an important role in inter-population dispersion, thus intensifying the dispersion of insecticide resistance associated- alleles as well as potentially mediating a quicker spread of arboviruses such as Dengue, Zika and Chikungunya to nearby municipalities.

## Conclusions

Our study showed that there is little differentiation among Sergipe’s *Ae. aegypti* population, possibly reflecting intense gene flow mediated by the passive dispersion of mosquitoes, due to the intense traffic of vehicles among the cities. The results of this work could provide important assistance in the performance assessment of strategies for the control and handling of *Ae. aegypti*. Such actions have been the basis for the prevention of Dengue fever, Zika virus and Chikungunya epidemics with a focus on the control of both larval and adult stages chiefly with insecticide. That practice has generated resistance processes, compromising mosquito control and increasing the risk of epidemics every year. Thus, knowledge of the genetic structuring and population dynamics of the species is crucial as these genetically different populations may present differences concerning their vectorial capacity and vector competence.

## Additional files

Additional file 1: Table S1. ISSR genetic diversity across all seven *Aedes aegypti* populations from Sergipe, Brazil. (PDF 305 kb)
Additional file 2: Figure S1. Profile of the bands amplified with ISSR molecular marker for 20 samples of *Aedes aegypti* of the population of Pinhão. (PDF 196 kb)
Additional file 3: Table S2. Summary of SNP markers variation in different Sergipe populations of *Aedes aegypti*. (PDF 123 kb)
Additional file 4: Figure S2. Number of alleles detected per locuspopulation by SNP genotyping in seven *Aedes aegypti* populations from Sergipe Brazil. (PDF 73 kb)
Additional file 5: Table S3. Chi-square test and probabilities applied to infer Hardy-Weinberg departures for seven loci in *Aedes aegypti* mosquitoes. (PDF 64 kb)
Additional file 6: Tables S4 and S5. Matrix of Nei (1972) genetic distance for Sergipe’s *Aedes aegypti* populations based on ISSR and SNP markers. (PDF 62 kb)
Additional file 7: Figure S3. Expected heterozygosity detected for seven SNP loci in *Aedes aegypti* populations. (PDF 74 kb)
Additional file 8: Figure S4. Clustering analysis using (A–D) Bayesian assignment of twenty runs for each K, K = 1 to K = 10. (PDF 90 kb)
Additional file 9: Figure S5. Pairwise relationship between genetic distance FST/(1- FST) vs geographical distance (km). (PDF 109 kb)

## Abbreviations

*aeimuc*: Mucin-like protein
*apolp-2*: Apolipophorin II
ARA: Aracaju
BIC: Bayesian information criterion
CA: Carira
*Chym*: Chymotrypsin
CSF: Canindé de São Francisco
*cyp9j2*: Cytochrome P450
DAPC: Discriminant analysis of principal components
dNTP: Deoxynucleotide
*ef2*: Elongation factor
*ferh*: Ferritin heavy chain
H_E_: Expected heterozygosity
HW: Hardy-Weinberg
ISSR: Inter-simple sequence repeat
LnP(D): Log likelihood
MA: Maruim
*nak*: Sodium/potassium channel
NEO: Neópolis
PCR: Polymerase chain reaction
*pgk*: Phosphoglycerate kinase
PI: Pinhão
R: Purine
RAPD: Random amplified polymorphic
RFLP: Restriction fragment length polymorphism
SNP: Single nucleotide polymorphism
SSCP: Single-strand conformation polymorphism
*tsf*: Transferrin
UMB: Umbaúba
Y: Pyrimidine
