# Supplementary material for: Genetic variability and spatial distribution in small geographic scale of Aedes aegypti (Diptera: Culicidae) under different climatic conditions in Northeastern Brazil
Source: Parasit Vectors. 2016 Oct 4;9:530. doi: 10.1186/s13071-016-1814-9 (PMC5050563; doi:10.1186/s13071-016-1814-9)
Supplement: Additional file 1: Table S1. — ISSR genetic diversity across all seven Aedes aegypti populations from Sergipe, Brazil. (PDF 305 kb) [file 13071_2016_1814_MOESM1_ESM.pdf]

Table S1. ISSR genetic diversity across all seven *Aedes aegypti* populations from Sergipe, Brazil.

Population	Locus	Band Freq.	p	q	N	Na	Ne	H_E
**CSF**	CA2230	0.000	0.000	1.000	19	0.0	1.000	0.000
	CA1850	0.684	0.438	0.562	19	2.0	1.970	0.492
	CA1750	0.579	0.351	0.649	19	2.0	1.837	0.456
	CA1600	0.053	0.027	0.973	19	2.0	1.055	0.052
	CA1500	0.053	0.027	0.973	19	2.0	1.055	0.052
	CA1400	0.368	0.205	0.795	19	2.0	1.484	0.326
	CA1200	1.000	1.000	0.000	19	1.0	1.000	0.000
	CA1150	0.000	0.000	1.000	19	0.0	1.000	0.000
	CA1050	0.368	0.205	0.795	19	2.0	1.484	0.326
	CA950	0.368	0.205	0.795	19	2.0	1.484	0.326
	CA900	0.000	0.000	1.000	19	0.0	1.000	0.000
	CA850	0.316	0.173	0.827	19	2.0	1.400	0.286
	CA800	0.263	0.142	0.858	19	2.0	1.321	0.243
	CA750	0.579	0.351	0.649	19	2.0	1.837	0.456
	CA600	0.789	0.541	0.459	19	2.0	1.987	0.497
	CA550	0.000	0.000	1.000	19	0.0	1.000	0.000
	CA450	0.158	0.082	0.918	19	2.0	1.178	0.151
	GA1700	0.474	0.275	0.725	19	2.0	1.662	0.398
	GA1500	0.789	0.541	0.459	19	2.0	1.987	0.497
	GA1350	0.368	0.205	0.795	19	2.0	1.484	0.326
	GA1050	0.211	0.111	0.889	19	2.0	1.247	0.198
	GA900	0.737	0.487	0.513	19	2.0	1.999	0.500
	GA850	0.632	0.393	0.607	19	2.0	1.912	0.477
	GA800	0.000	0.000	1.000	19	0.0	1.000	0.000
	GA650	0.526	0.312	0.688	19	2.0	1.752	0.429
	GA550	0.789	0.541	0.459	19	2.0	1.987	0.497
	GA500	0.421	0.239	0.761	19	2.0	1.572	0.364
	GA450	0.632	0.393	0.607	19	2.0	1.912	0.477
	GA400	0.000	0.000	1.000	19	0.0	1.000	0.000
	GA350	0.000	0.000	1.000	19	0.0	1.000	0.000
**CA**	CA2230	0.000	0.000	1.000	20	0.0	1.000	0.000
	CA1850	0.450	0.258	0.742	20	2.0	1.621	0.383
	CA1750	0.050	0.025	0.975	20	2.0	1.052	0.049

	CA1600	0.050	0.025	0.975	20	2.0	1.052	0.049
	CA1500	0.000	0.000	1.000	20	0.0	1.000	0.000
	CA1400	0.400	0.225	0.775	20	2.0	1.537	0.349
	CA1200	1.000	1.000	0.000	20	1.0	1.000	0.000
	CA1150	0.100	0.051	0.949	20	2.0	1.108	0.097
	CA1050	0.900	0.684	0.316	20	2.0	1.762	0.432
	CA950	0.000	0.000	1.000	20	0.0	1.000	0.000
	CA900	0.200	0.106	0.894	20	2.0	1.233	0.189
	CA850	0.300	0.163	0.837	20	2.0	1.376	0.273
	CA800	0.200	0.106	0.894	20	2.0	1.233	0.189
	CA750	0.200	0.106	0.894	20	2.0	1.233	0.189
	CA600	0.400	0.225	0.775	20	2.0	1.537	0.349
	CA550	0.000	0.000	1.000	20	0.0	1.000	0.000
	CA450	0.400	0.225	0.775	20	2.0	1.537	0.349
	GA1700	0.100	0.051	0.949	20	2.0	1.108	0.097
	GA1500	0.200	0.106	0.894	20	2.0	1.233	0.189
	GA1350	0.050	0.025	0.975	20	2.0	1.052	0.049
	GA1050	0.100	0.051	0.949	20	2.0	1.108	0.097
	GA900	0.600	0.368	0.632	20	2.0	1.869	0.465
	GA850	0.500	0.293	0.707	20	2.0	1.707	0.414
	GA800	0.250	0.134	0.866	20	2.0	1.302	0.232
	GA650	0.850	0.613	0.387	20	2.0	1.903	0.475
	GA550	1.000	1.000	0.000	20	1.0	1.000	0.000
	GA500	0.000	0.000	1.000	20	0.0	1.000	0.000
	GA450	0.900	0.684	0.316	20	2.0	1.762	0.432
	GA400	0.200	0.106	0.894	20	2.0	1.233	0.189
	GA350	0.650	0.408	0.592	20	2.0	1.935	0.483
**MA**	CA2230	0.182	0.095	0.905	22	2.0	1.209	0.173
	CA1850	0.091	0.047	0.953	22	2.0	1.097	0.089
	CA1750	0.591	0.360	0.640	22	2.0	1.855	0.461
	CA1600	0.000	0.000	1.000	22	0.0	1.000	0.000
	CA1500	0.000	0.000	1.000	22	0.0	1.000	0.000
	CA1400	0.091	0.047	0.953	22	2.0	1.097	0.089
	CA1200	1.000	1.000	0.000	22	1.0	1.000	0.000
	CA1150	0.000	0.000	1.000	22	0.0	1.000	0.000
	CA1050	0.591	0.360	0.640	22	2.0	1.855	0.461
	CA950	0.364	0.202	0.798	22	2.0	1.476	0.323
	CA900	0.000	0.000	1.000	22	0.0	1.000	0.000
	CA850	0.227	0.121	0.879	22	2.0	1.270	0.213
	CA800	0.000	0.000	1.000	22	0.0	1.000	0.000
	CA750	0.000	0.000	1.000	22	0.0	1.000	0.000
	CA600	0.773	0.523	0.477	22	2.0	1.996	0.499

	CA550	0.000	0.000	1.000	22	0.0	1.000	0.000
	CA450	0.000	0.000	1.000	22	0.0	1.000	0.000
	GA1700	0.045	0.023	0.977	22	2.0	1.047	0.045
	GA1500	1.000	1.000	0.000	22	1.0	1.000	0.000
	GA1350	0.000	0.000	1.000	22	0.0	1.000	0.000
	GA1050	0.273	0.147	0.853	22	2.0	1.335	0.251
	GA900	0.773	0.523	0.477	22	2.0	1.996	0.499
	GA850	0.545	0.326	0.674	22	2.0	1.784	0.439
	GA800	0.000	0.000	1.000	22	0.0	1.000	0.000
	GA650	0.591	0.360	0.640	22	2.0	1.855	0.461
	GA550	0.727	0.478	0.522	22	2.0	1.996	0.499
	GA500	0.000	0.000	1.000	22	0.0	1.000	0.000
	GA450	0.136	0.071	0.929	22	2.0	1.151	0.131
	GA400	0.000	0.000	1.000	22	0.0	1.000	0.000
	GA350	0.000	0.000	1.000	22	0.0	1.000	0.000
**PI**	CA2230	0.000	0.000	1.000	20	0.0	1.000	0.000
	CA1850	0.900	0.684	0.316	20	2.0	1.762	0.432
	CA1750	0.100	0.051	0.949	20	2.0	1.108	0.097
	CA1600	0.000	0.000	1.000	20	0.0	1.000	0.000
	CA1500	0.100	0.051	0.949	20	2.0	1.108	0.097
	CA1400	0.400	0.225	0.775	20	2.0	1.537	0.349
	CA1200	1.000	1.000	0.000	20	1.0	1.000	0.000
	CA1150	0.000	0.000	1.000	20	0.0	1.000	0.000
	CA1050	0.750	0.500	0.500	20	2.0	2.000	0.500
	CA950	0.150	0.078	0.922	20	2.0	1.168	0.144
	CA900	0.000	0.000	1.000	20	0.0	1.000	0.000
	CA850	0.000	0.000	1.000	20	0.0	1.000	0.000
	CA800	0.200	0.106	0.894	20	2.0	1.233	0.189
	CA750	0.000	0.000	1.000	20	0.0	1.000	0.000
	CA600	0.900	0.684	0.316	20	2.0	1.762	0.432
	CA550	0.000	0.000	1.000	20	0.0	1.000	0.000
	CA450	0.000	0.000	1.000	20	0.0	1.000	0.000
	GA1700	0.000	0.000	1.000	20	0.0	1.000	0.000
	GA1500	1.000	1.000	0.000	20	1.0	1.000	0.000
	GA1350	0.000	0.000	1.000	20	0.0	1.000	0.000
	GA1050	0.600	0.368	0.632	20	2.0	1.869	0.465
	GA900	0.200	0.106	0.894	20	2.0	1.233	0.189
	GA850	0.900	0.684	0.316	20	2.0	1.762	0.432
	GA800	0.100	0.051	0.949	20	2.0	1.108	0.097
	GA650	0.200	0.106	0.894	20	2.0	1.233	0.189
	GA550	1.000	1.000	0.000	20	1.0	1.000	0.000
	GA500	0.000	0.000	1.000	20	0.0	1.000	0.000

	GA450	0.000	0.000	1.000	20	0.0	1.000	0.000
	GA400	0.000	0.000	1.000	20	0.0	1.000	0.000
	GA350	0.000	0.000	1.000	20	0.0	1.000	0.000
**PD**	CA2230	0.000	0.000	1.000	19	0.0	1.000	0.000
	CA1850	0.789	0.541	0.459	19	2.0	1.987	0.497
	CA1750	0.421	0.239	0.761	19	2.0	1.572	0.364
	CA1600	0.000	0.000	1.000	19	0.0	1.000	0.000
	CA1500	0.105	0.054	0.946	19	2.0	1.114	0.102
	CA1400	0.421	0.239	0.761	19	2.0	1.572	0.364
	CA1200	1.000	1.000	0.000	19	1.0	1.000	0.000
	CA1150	0.000	0.000	1.000	19	0.0	1.000	0.000
	CA1050	0.579	0.351	0.649	19	2.0	1.837	0.456
	CA950	0.789	0.541	0.459	19	2.0	1.987	0.497
	CA900	0.316	0.173	0.827	19	2.0	1.400	0.286
	CA850	0.316	0.173	0.827	19	2.0	1.400	0.286
	CA800	0.474	0.275	0.725	19	2.0	1.662	0.398
	CA750	0.263	0.142	0.858	19	2.0	1.321	0.243
	CA600	0.789	0.541	0.459	19	2.0	1.987	0.497
	CA550	0.158	0.082	0.918	19	2.0	1.178	0.151
	CA450	0.000	0.000	1.000	19	0.0	1.000	0.000
	GA1700	0.000	0.000	1.000	19	0.0	1.000	0.000
	GA1500	1.000	1.000	0.000	19	1.0	1.000	0.000
	GA1350	0.000	0.000	1.000	19	0.0	1.000	0.000
	GA1050	0.211	0.111	0.889	19	2.0	1.247	0.198
	GA900	1.000	1.000	0.000	19	1.0	1.000	0.000
	GA850	0.632	0.393	0.607	19	2.0	1.912	0.477
	GA800	0.737	0.487	0.513	19	2.0	1.999	0.500
	GA650	0.842	0.603	0.397	19	2.0	1.919	0.479
	GA550	0.895	0.676	0.324	19	2.0	1.780	0.438
	GA500	0.105	0.054	0.946	19	2.0	1.114	0.102
	GA450	0.579	0.351	0.649	19	2.0	1.837	0.456
	GA400	0.000	0.000	1.000	19	0.0	1.000	0.000
	GA350	0.000	0.000	1.000	19	0.0	1.000	0.000
**NEO**	CA2230	0.000	0.000	1.000	20	0.0	1.000	0.000
	CA1850	0.700	0.452	0.548	20	2.0	1.982	0.495
	CA1750	0.000	0.000	1.000	20	0.0	1.000	0.000
	CA1600	0.100	0.051	0.949	20	2.0	1.108	0.097
	CA1500	0.000	0.000	1.000	20	0.0	1.000	0.000
	CA1400	0.050	0.025	0.975	20	2.0	1.052	0.049
	CA1200	1.000	1.000	0.000	20	1.0	1.000	0.000
	CA1150	0.000	0.000	1.000	20	0.0	1.000	0.000
	CA1050	0.450	0.258	0.742	20	2.0	1.621	0.383

	CA950	0.600	0.368	0.632	20	2.0	1.869	0.465
	CA900	0.000	0.000	1.000	20	0.0	1.000	0.000
	CA850	0.350	0.194	0.806	20	2.0	1.454	0.312
	CA800	0.150	0.078	0.922	20	2.0	1.168	0.144
	CA750	0.200	0.106	0.894	20	2.0	1.233	0.189
	CA600	0.500	0.293	0.707	20	2.0	1.707	0.414
	CA550	0.150	0.078	0.922	20	2.0	1.168	0.144
	CA450	0.000	0.000	1.000	20	0.0	1.000	0.000
	GA1700	0.050	0.025	0.975	20	2.0	1.052	0.049
	GA1500	1.000	1.000	0.000	20	1.0	1.000	0.000
	GA1350	0.000	0.000	1.000	20	0.0	1.000	0.000
	GA1050	0.300	0.163	0.837	20	2.0	1.376	0.273
	GA900	0.450	0.258	0.742	20	2.0	1.621	0.383
	GA850	0.950	0.776	0.224	20	2.0	1.532	0.347
	GA800	0.000	0.000	1.000	20	0.0	1.000	0.000
	GA650	0.700	0.452	0.548	20	2.0	1.982	0.495
	GA550	0.900	0.684	0.316	20	2.0	1.762	0.432
	GA500	0.000	0.000	1.000	20	0.0	1.000	0.000
	GA450	0.550	0.329	0.671	20	2.0	1.791	0.442
	GA400	0.000	0.000	1.000	20	0.0	1.000	0.000
	GA350	0.000	0.000	1.000	20	0.0	1.000	0.000
**UMB**	CA2230	0.263	0.142	0.858	19	2.0	1.321	0.243
	CA1850	0.158	0.082	0.918	19	2.0	1.178	0.151
	CA1750	0.368	0.205	0.795	19	2.0	1.484	0.326
	CA1600	0.000	0.000	1.000	19	0.0	1.000	0.000
	CA1500	0.000	0.000	1.000	19	0.0	1.000	0.000
	CA1400	0.316	0.173	0.827	19	2.0	1.400	0.286
	CA1200	1.000	1.000	0.000	19	1.0	1.000	0.000
	CA1150	0.000	0.000	1.000	19	0.0	1.000	0.000
	CA1050	0.316	0.173	0.827	19	2.0	1.400	0.286
	CA950	0.000	0.000	1.000	19	0.0	1.000	0.000
	CA900	0.211	0.111	0.889	19	2.0	1.247	0.198
	CA850	0.158	0.082	0.918	19	2.0	1.178	0.151
	CA800	0.263	0.142	0.858	19	2.0	1.321	0.243
	CA750	0.526	0.312	0.688	19	2.0	1.752	0.429
	CA600	0.947	0.771	0.229	19	2.0	1.547	0.354
	CA550	0.842	0.603	0.397	19	2.0	1.919	0.479
	CA450	0.000	0.000	1.000	19	0.0	1.000	0.000
	GA1700	0.000	0.000	1.000	19	0.0	1.000	0.000
	GA1500	0.000	0.000	1.000	19	0.0	1.000	0.000
	GA1350	0.000	0.000	1.000	19	0.0	1.000	0.000
	GA1050	0.211	0.111	0.889	19	2.0	1.247	0.198

GA900	0.526	0.312	0.688	19	2.0	1.752	0.429
GA850	0.316	0.173	0.827	19	2.0	1.400	0.286
GA800	0.158	0.082	0.918	19	2.0	1.178	0.151
GA650	0.684	0.438	0.562	19	2.0	1.970	0.492
GA550	0.895	0.676	0.324	19	2.0	1.780	0.438
GA500	0.263	0.142	0.858	19	2.0	1.321	0.243
GA450	0.368	0.205	0.795	19	2.0	1.484	0.326
GA400	0.000	0.000	1.000	19	0.0	1.000	0.000
GA350	0.000	0.000	1.000	19	0.0	1.000	0.000

N; Sample size, Na; Number of Different Alleles, Ne; Number of Effective Alleles, $H_{E;}$ Expected Heterozygosity
