# Supplementary material for: Genetic variability and spatial distribution in small geographic scale of Aedes aegypti (Diptera: Culicidae) under different climatic conditions in Northeastern Brazil
Source: Parasit Vectors. 2016 Oct 4;9:530. doi: 10.1186/s13071-016-1814-9 (PMC5050563; doi:10.1186/s13071-016-1814-9)
Supplement: Additional file 2: Figure S1. — Profile of the bands amplified with ISSR molecular marker for 20 samples of Aedes aegypti of the population of Pinhão. (PDF 196 kb) [file 13071_2016_1814_MOESM2_ESM.pdf]

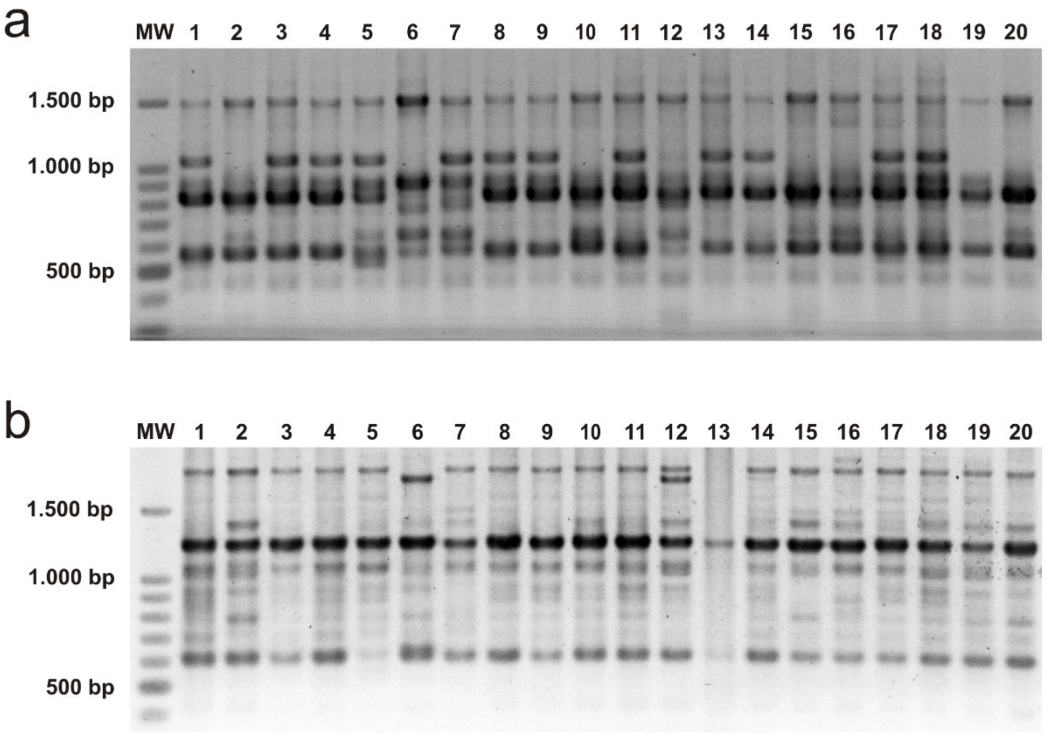

Figure S1: Profile of the bands amplified with ISSR molecular marker for 20 samples of *Aedes aegypti* of the population of Pinhão. a) band profile for the initiator $(GA)_8RY$. b) band profile for the initiator $(CA)_8RY$. MW = Molecular Weight.
