# Supplementary material for: Genetic variability and spatial distribution in small geographic scale of Aedes aegypti (Diptera: Culicidae) under different climatic conditions in Northeastern Brazil
Source: Parasit Vectors. 2016 Oct 4;9:530. doi: 10.1186/s13071-016-1814-9 (PMC5050563; doi:10.1186/s13071-016-1814-9)
Supplement: Additional file 3: Table S2. — Summary of SNP markers variation in different Sergipe populations of Aedes aegypti. (PDF 123 kb) [file 13071_2016_1814_MOESM3_ESM.pdf]

Table S2. Summary of SNP markers variation in different Sergipe populations of *Ae. aegypti*.

Population	Locus	N	Na	Ne	H_O	H_E	uHe	F
**CSF**	EF2	20	2.000	1.600	0.400	0.375	0.385	-0.067
	MUC	20	2.000	1.923	0.500	0.480	0.492	-0.042
	NAK	20	2.000	1.161	0.150	0.139	0.142	-0.081
	PGK	20	2.000	1.536	0.350	0.349	0.358	-0.004
	APOL	20	2.000	1.471	0.400	0.320	0.328	-0.250
	FERR	20	2.000	1.835	0.500	0.455	0.467	-0.099
	P450	20	2.000	2.000	0.700	0.500	0.513	-0.400
	TSF	20	2.000	1.220	0.200	0.180	0.185	-0.111
	CHYM	20	2.000	1.835	0.400	0.455	0.467	0.121
**CA**	EF2	20	2.000	1.406	0.350	0.289	0.296	-0.212
	MUC	20	2.000	2.000	0.600	0.500	0.513	-0.200
	NAK	20	2.000	1.105	0.100	0.095	0.097	-0.053
	PGK	20	1.000	1.000	0.000	0.000	0.000	N/A*
	APOL	20	2.000	1.995	0.150	0.499	0.512	0.699
	FERR	19	2.000	1.951	0.316	0.488	0.501	0.352
	P450	20	2.000	1.782	0.450	0.439	0.450	-0.026
	TSF	20	2.000	1.105	0.100	0.095	0.097	-0.053
	CHYM	20	2.000	1.956	0.350	0.489	0.501	0.284
**PI**	EF2	19	2.000	1.870	0.632	0.465	0.478	-0.357
	MUC	18	2.000	1.857	0.278	0.461	0.475	0.398
	NAK	20	1.000	1.000	0.000	0.000	0.000	N/A*
	PGK	20	2.000	1.161	0.150	0.139	0.142	-0.081
	APOL	20	2.000	1.782	0.550	0.439	0.450	-0.254
	FERR	20	2.000	1.882	0.750	0.469	0.481	-0.600
	P450	20	2.000	1.923	0.500	0.480	0.492	-0.042
	TSF	20	2.000	1.471	0.400	0.320	0.328	-0.250
	CHYM	20	2.000	1.980	0.600	0.495	0.508	-0.212
**MA**	EF2	20	2.000	1.663	0.350	0.399	0.409	0.122
	MUC	19	2.000	1.566	0.263	0.361	0.371	0.272
	NAK	20	2.000	1.280	0.250	0.219	0.224	-0.143
	PGK	20	2.000	1.161	0.150	0.139	0.142	-0.081
	APOL	20	2.000	1.782	0.550	0.439	0.450	-0.254
	FERR	20	2.000	1.536	0.450	0.349	0.358	-0.290
	P450	20	2.000	1.471	0.300	0.320	0.328	0.062
	TSF	20	2.000	1.280	0.250	0.219	0.224	-0.143
	CHYM	20	2.000	1.882	0.450	0.469	0.481	0.040
**ARA**	EF2	20	2.000	1.536	0.350	0.349	0.358	-0.004
	MUC	18	2.000	1.670	0.444	0.401	0.413	-0.108
	NAK	19	2.000	1.111	0.105	0.100	0.102	-0.056

	PGK	20	2.000	1.161	0.150	0.139	0.142	-0.081
	APOL	20	2.000	1.923	0.400	0.480	0.492	0.167
	FERR	20	2.000	1.663	0.350	0.399	0.409	0.122
	P450	16	2.000	1.822	0.313	0.451	0.466	0.307
	TSF	20	2.000	1.161	0.150	0.139	0.142	-0.081
	CHYM	20	2.000	1.663	0.450	0.399	0.409	-0.129
**NEO**	EF2	20	2.000	1.342	0.200	0.255	0.262	0.216
	MUC	20	2.000	1.342	0.200	0.255	0.262	0.216
	NAK	20	2.000	1.342	0.100	0.255	0.262	0.608
	PGK	20	2.000	1.280	0.250	0.219	0.224	-0.143
	APOL	20	2.000	1.835	0.600	0.455	0.467	-0.319
	FERR	20	1.000	1.000	0.000	0.000	0.000	N/A*
	P450	20	2.000	1.342	0.100	0.255	0.262	0.608
	TSF	20	2.000	1.536	0.250	0.349	0.358	0.283
	CHYM	20	2.000	1.600	0.100	0.375	0.385	0.733
**UMB**	EF2	20	2.000	1.782	0.650	0.439	0.450	-0.481
	MUC	19	2.000	1.870	0.211	0.465	0.478	0.548
	NAK	20	2.000	1.105	0.100	0.095	0.097	-0.053
	PGK	20	2.000	1.663	0.550	0.399	0.409	-0.379
	APOL	20	2.000	2.000	0.700	0.500	0.513	-0.400
	FERR	20	2.000	1.782	0.350	0.439	0.450	0.202
	P450	20	2.000	1.980	0.500	0.495	0.508	-0.010
	TSF	20	2.000	1.105	0.100	0.095	0.097	-0.053
	CHYM	20	2.000	1.835	0.300	0.455	0.467	0.341

N; Sample size, Na; Number of Different Alleles, Ne; Number of Effective Alleles, $H_{O;}$ Observed Heterozygosity, $H_{E;}$ Expected Heterozygosity, F; Fixation Index.
*Not
