# Supplementary material for: Genetic variability and spatial distribution in small geographic scale of Aedes aegypti (Diptera: Culicidae) under different climatic conditions in Northeastern Brazil
Source: Parasit Vectors. 2016 Oct 4;9:530. doi: 10.1186/s13071-016-1814-9 (PMC5050563; doi:10.1186/s13071-016-1814-9)
Supplement: Additional file 4: Figure S2. — Number of alleles detected per locuspopulation by SNP genotyping in seven Aedes aegypti populations from Sergipe Brazil. (PDF 73 kb) [file 13071_2016_1814_MOESM4_ESM.pdf]

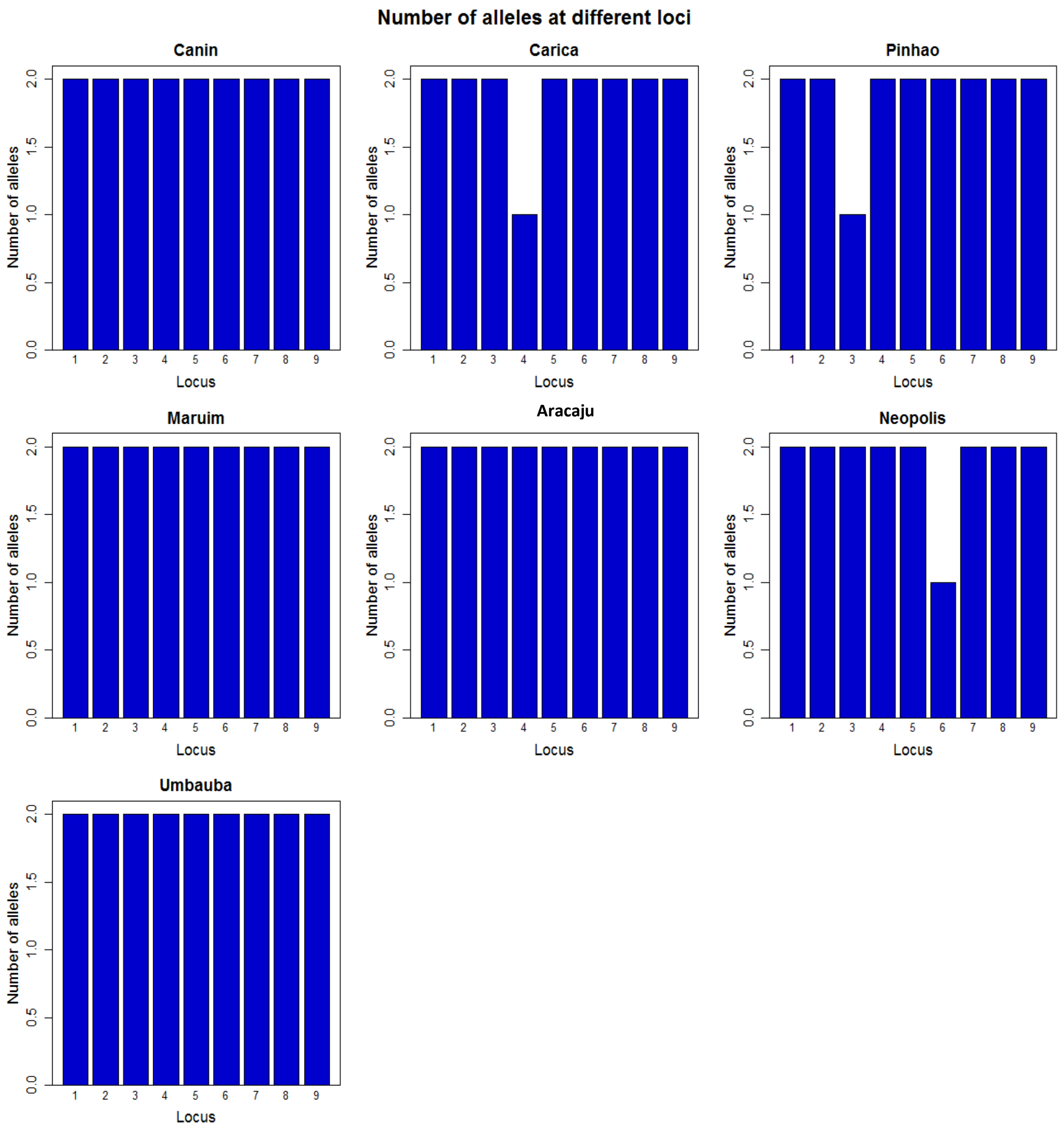

Figure S2. Number of alleles detected for nine loci by SNP genotyping in seven *Aedes aegypti* populations from Sergipe Brazil.
