# Supplementary material for: Genetic variability and spatial distribution in small geographic scale of Aedes aegypti (Diptera: Culicidae) under different climatic conditions in Northeastern Brazil
Source: Parasit Vectors. 2016 Oct 4;9:530. doi: 10.1186/s13071-016-1814-9 (PMC5050563; doi:10.1186/s13071-016-1814-9)
Supplement: Additional file 5: Table S3. — Chi-square test and probabilities applied to infer Hardy-Weinberg departures for seven loci in Aedes aegypti mosquitoes. (PDF 64 kb) [file 13071_2016_1814_MOESM5_ESM.pdf]

Table S3. Chi-squared test and probabilities applied to infer Hardy-Weinberg departures for seven loci in *Ae. aegypti* mosquitoes.

	Population													
**Locus**	**CSF**		**CA**		**PI**		**MA**		**ARA**		**NEO**		**UMB**	
EF2	0.089	(0.766^{NS})	0.900	(0.343^{NS})	2.423	(0.120^{NS})	0.299	(0.585^{NS})	0.000	(0.987^{NS})	0.930	(0.335^{NS})	4.636	(0.031**)
MUC	0.035	(0.852^{NS})	0.800	(0.371^{NS})	2.851	(0.091^{NS})	1.406	(0.236^{NS})	0.209	(0.648^{NS})	0.930	(0.335^{NS})	5.698	(0.017**)
NAK	0.131	(0.717^{NS})	0.055	(0.814^{NS})	Monomorphic		0.408	(0.523^{NS})	0.059	(0.809^{NS})	7.389	(0.007**)	0.055	(0.814^{NS})
PGK	0.000	(0.987^{NS})	Monomorphic		0.131	(0.717^{NS})	0.131	(0.717^{NS})	0.131	(0.717^{NS})	0.408	(0.523^{NS})	2.878	(0.090^{NS})
APOL	1.250	(0.264^{NS})	9.779	(0.002**)	1.286	(0.257^{NS})	1.286	(0.257^{NS})	0.556	(0.456^{NS})	2.031	(0.154^{NS})	3.200	(0.074^{NS})
FerH	0.196	(0.658^{NS})	2.358	(0.125^{NS})	7.200	(0.007**)	1.686	(0.194^{NS})	0.299	(0.585^{NS})	Monomorphic		0.818	(0.366^{NS})
P450	3.200	(0.074^{NS})	0.013	(0.909^{NS})	0.035	(0.852^{NS})	0.078	(0.780^{NS})	1.512	(0.219^{NS})	7.389	(0.007^{NS})	0.002	(0.964^{NS})
TSF	0.247	(0.619^{NS})	0.055	(0.814^{NS})	1.250	(0.264^{NS})	0.408	(0.523^{NS})	0.131	(0.717^{NS})	1.604	(0.205^{NS})	0.055	(0.814^{NS})
CHYM	0.292	(0.589^{NS})	1.612	(0.204^{NS})	0.900	(0.343^{NS})	0.032	(0.858^{NS})	0.330	(0.565^{NS})	10.756	(0.001**)	2.321	(0.128^{NS})

Key: ns=not significant (P > 0.05) , ** P<0.01
