# Supplementary material for: Genetic variability and spatial distribution in small geographic scale of Aedes aegypti (Diptera: Culicidae) under different climatic conditions in Northeastern Brazil
Source: Parasit Vectors. 2016 Oct 4;9:530. doi: 10.1186/s13071-016-1814-9 (PMC5050563; doi:10.1186/s13071-016-1814-9)
Supplement: Additional file 6: Tables S4 and S5. — Matrix of Nei (1972) genetic distance for Sergipe’s Aedes aegypti populations based on ISSR and SNP markers. (PDF 62 kb) [file 13071_2016_1814_MOESM6_ESM.pdf]

Table S4. Matrix of Nei (1972) genetic distance for Sergipe`s *Aedes aegypti* populations based on ISSR markers

	**CSF**	**CA**	**MA**	**PI**	**ARA**	**NEO**	**UMB**
**CSF**	0.000						
**CA**	0.069	0.000					
**MA**	0.034	0.096	0.000				
**PI**	0.061	0.107	0.053	0.000			
**ARA**	0.056	0.110	0.050	0.084	0.000		
**NEO**	0.039	0.084	0.033	0.034	0.053	0.000	
**UMB**	0.054	0.074	0.077	0.110	0.120	0.101	0.000

Table S5. Matrix of Nei (1972) genetic distance for Sergipe`s *Aedes aegypti* populations based on SNP markers

	**CSF**	**CA**	**PI**	**MA**	**ARA**	**NEO**	**UMB**
**CSF**	0.000						
**CA**	0.037	0.000					
**PI**	0.039	0.053	0.000				
**MA**	0.086	0.029	0.088	0.000			
**ARA**	0.055	0.018	0.075	0.008	0.000		
**NEO**	0.118	0.070	0.145	0.016	0.027	0.000	
**UMB**	0.045	0.032	0.056	0.043	0.038	0.086	0.000
