# Supplementary material for: Genetic variability and spatial distribution in small geographic scale of Aedes aegypti (Diptera: Culicidae) under different climatic conditions in Northeastern Brazil
Source: Parasit Vectors. 2016 Oct 4;9:530. doi: 10.1186/s13071-016-1814-9 (PMC5050563; doi:10.1186/s13071-016-1814-9)
Supplement: Additional file 8: Figure S4. — Clustering analysis using (A–D) Bayesian assignment of twenty runs for each K, K = 1 to K = 10. (PDF 90 kb) [file 13071_2016_1814_MOESM8_ESM.pdf]

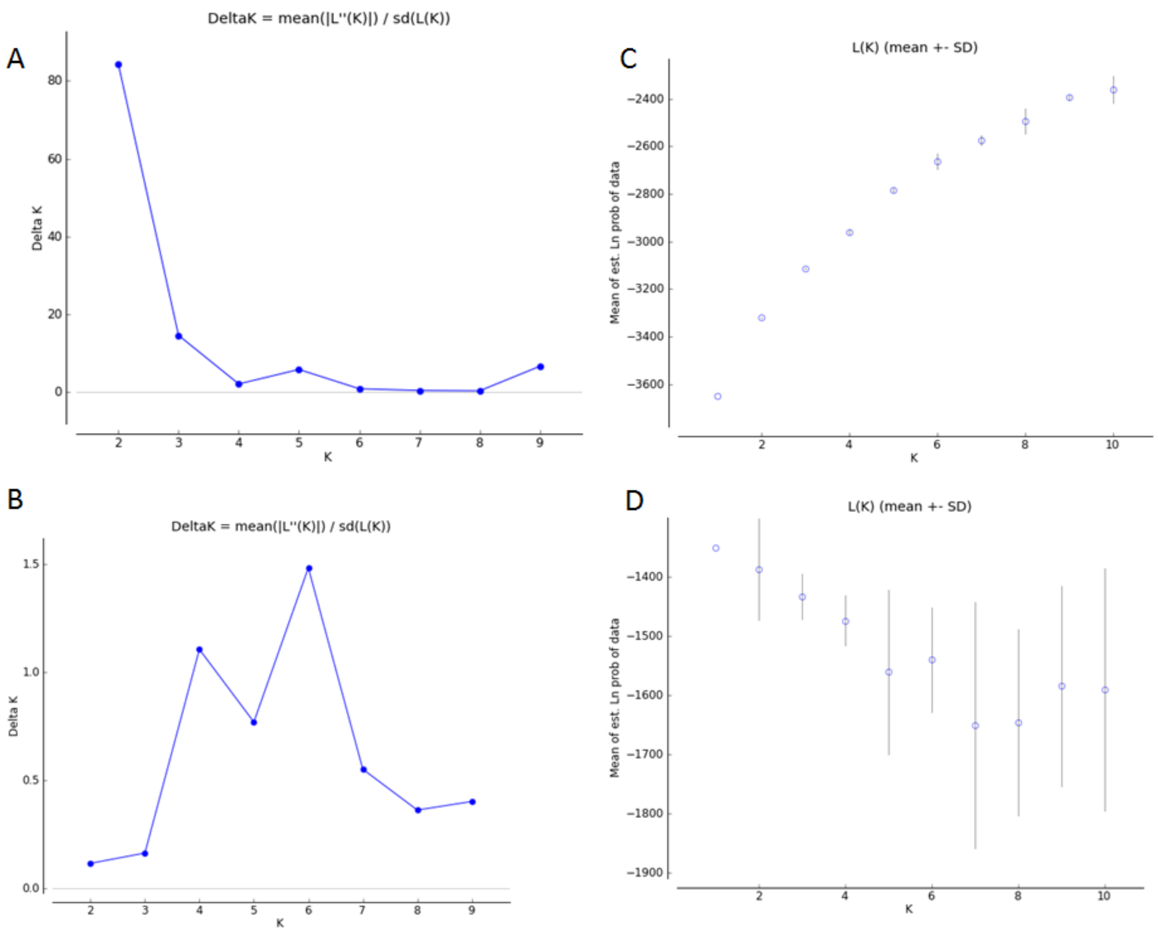

Figure S4. Clustering analysis using (*A*–*D*) Bayesian assignment of twenty runs for each K, *K* = 1 to *K* = 10. **A** and **B)** Delta *K* results for the optimal value of *K* for structure analysis based on ISSR and SNP markers, respectively. **C** and **D)** Mean likelihood (*L*(*K*) (±s.d.)) dividing the entire dataset into *K* populations using ISSR and SNP markers, respectively.
