# Supplementary material for: Genetic variability and spatial distribution in small geographic scale of Aedes aegypti (Diptera: Culicidae) under different climatic conditions in Northeastern Brazil
Source: Parasit Vectors. 2016 Oct 4;9:530. doi: 10.1186/s13071-016-1814-9 (PMC5050563; doi:10.1186/s13071-016-1814-9)
Supplement: Additional file 9: Figure S5. — Pairwise relationship between genetic distance FST/(1- FST) vs geographical distance (km). (PDF 109 kb) [file 13071_2016_1814_MOESM9_ESM.pdf]

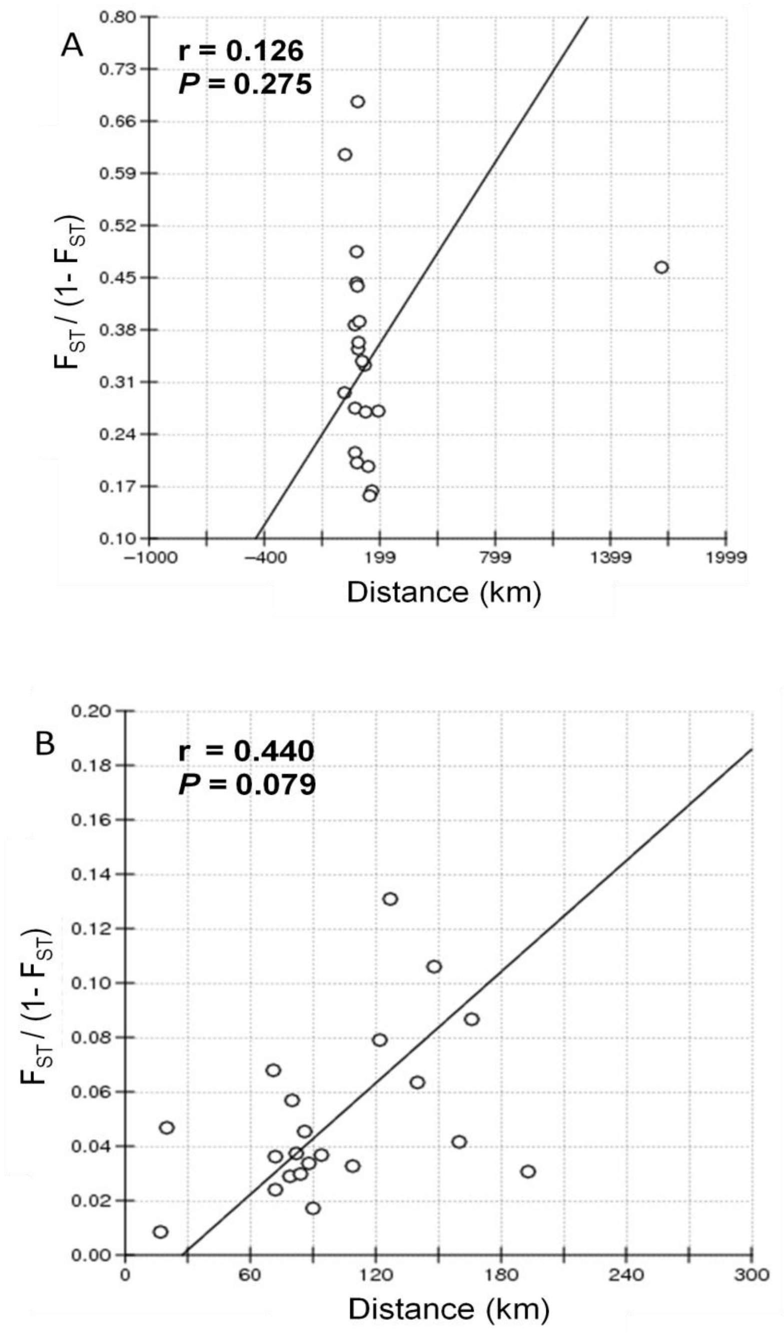

Figure S5. Pairwise relationship between genetic distance F_{ST} / (1- F_{ST}) vs. geographical distance (km). **A** and **B**) Test of isolation by distance using ISSR and SNP data, respectively.
